# Prevalence, sequence diversity, and amplification of an IS-associated enterotoxin gene, *astA*, in *Escherichia coli*

**DOI:** 10.3389/fmicb.2025.1635769

**Published:** 2025-10-22

**Authors:** Tadasuke Ooka, Sakura Arai, Kenichi Lee, Yasuhiro Gotoh, Akiko Kubomura, Naoko Imuta, Yukiko Hara-Kudo, Sunao Iyoda, Tetsuya Hayashi, Junichiro Nishi

**Affiliations:** ^1^Department of Microbiology, Graduate School of Medical and Dental Sciences, Kagoshima University, Kagoshima, Japan; ^2^Division of Microbiology, National Institute of Health Sciences, Kawasaki, Kanagawa, Japan; ^3^Department of Bacteriology I, National Institute of Infectious Diseases, Tokyo, Japan; ^4^Department of Bacteriology, Faculty of Medical Sciences, Kyushu University, Fukuoka, Japan; ^5^Advanced Genomics Center, National Institute of Genetics, Shizuoka, Japan; ^6^Department of Microbiology, Hoshi University, Tokyo, Japan

**Keywords:** *Escherichia coli*, enterotoxin EAST1, *astA* variant, IS1414, genotyping, pathogenesis

## Abstract

**Introduction:**

Enteroaggregative *Escherichia coli* heat-stable enterotoxin 1 (EAST1) encoded by the astA gene was first identified in an enteroaggregative *E. coli* strain isolated from a patient with persistent diarrhea. While astA-positive strains sometimes cause large food poisoning outbreaks, the significance of EAST1 as a virulence factor remains unclear. Additionally, although the prototype and seven variants of the *astA* gene have been identified, the biological significance of these genetic variations remains undefined. This study aimed to elucidate the characteristics of the A gene by investigating its distribution and sequence diversity within the evolutionary lineages of Escherichia coli.

**Methods:**

We conducted PCR screening for the *astA* gene in 2,726 *E. coli* strains isolated from children with diarrhea in Kagoshima, Japan, and blastn search of the *astA* gene was conducted on 9,065 publicly available finished *E. coli* genomes. The *astA* gene identified were subjected to analysis of sequence variation and comparison of their flanking genomic regions. In addition, the phylogenetic distribution of *astA* gene variants in *E. coli* lineage was also investigated.

**Results and discussion:**

The results showed that 185 (6.8%) of the Kagoshima strains and 690 (7.6%) of the database strains had similar possession rates. We identified 31 sequence variations (four known and 27 new variants [V8-34]) which were widely distributed in the *E. coli* lineages. Detailed sequence analyses revealed that 31 of the 35 *astA* gene types are intact and encode 23 types of EAST1 peptides. Although all 35 types were associated with IS*1414*, only three (prototype, V30, and V31) of the 31 intact *astA* gene types were encoded in the intact IS*1414*. A notable number of prototype-bearing strains (43/146 strains) possessed multiple copies (two to 11 copies) of this type of *astA* gene, indicating that the amplification has predominantly occurred in the prototype, which was driven by IS1414 amplification. However, given that the IS1414 associated with V30 and V31 also remain structurally intact, it is plausible that similar amplification events may occur in these variants in the future. These results provide an important basis to investigate the virulence of the *astA*-positive strains and the role of EAST1 as a virulence factor.

## Introduction

Enteroaggregative *Escherichia coli* heat-stable enterotoxin1 (EAST1), a small peptide (38 amino acids of 4.1 kDa) encoded by the *astA* gene, was first discovered in an enteroaggregative *E. coli* (EAggEC) strain 17-2 (Savarino et al., 1991; Ménard et al., 2004). The *astA* gene is known to be widely distributed in a variety of pathogenic *E. coli* strains (Yamamoto and Echeverria, 1996; Savarino et al., 1996; Paiva de Sousa and Dubreuil, 2001; Ménard and Dubreuil, 2002; Beutin et al., 2008; Maluta et al., 2017; Paniagua-Contreras et al., 2017). EAST1 shares 50% amino acid sequence identity with the enterotoxic domain of heat-stable enterotoxin (STa) and is proposed to exhibit similar mechanism of action to that of STa, which elicits an increase in cGMP on intestinal epithelial cells and subsequent fluid secretion (Dubreuil, 2019). Functional studies using Ussing chamber assays in rabbit ileal mucosa, as well as human T84 epithelial cell monolayers, have demonstrated that EAST1 can stimulate chloride ion secretion, evidenced by sustained increases in short-circuit current (Savarino et al., 1991; Veilleux et al., 2008). These findings support its potential role in the onset of diarrhea. On the other hand, the role of EAST1 in diarrhea *in vivo* is still questioned because some volunteers challenged with EAST1-producing EAEC strains did not develop diarrhea, even when the strains effectively colonized the intestine (Nataro et al., 1995). However, large-scale food poisoning outbreaks occurred in Japan by *E. coli* strains of serotypes O7:H4 and O166:H5, in which only the *astA* gene was detected as a potential virulence-related gene (Zhou et al., 2002; Kashima et al., 2021), suggesting that a possible contribution of EAST1 to the onset of diarrhea.

**Figure 1:**
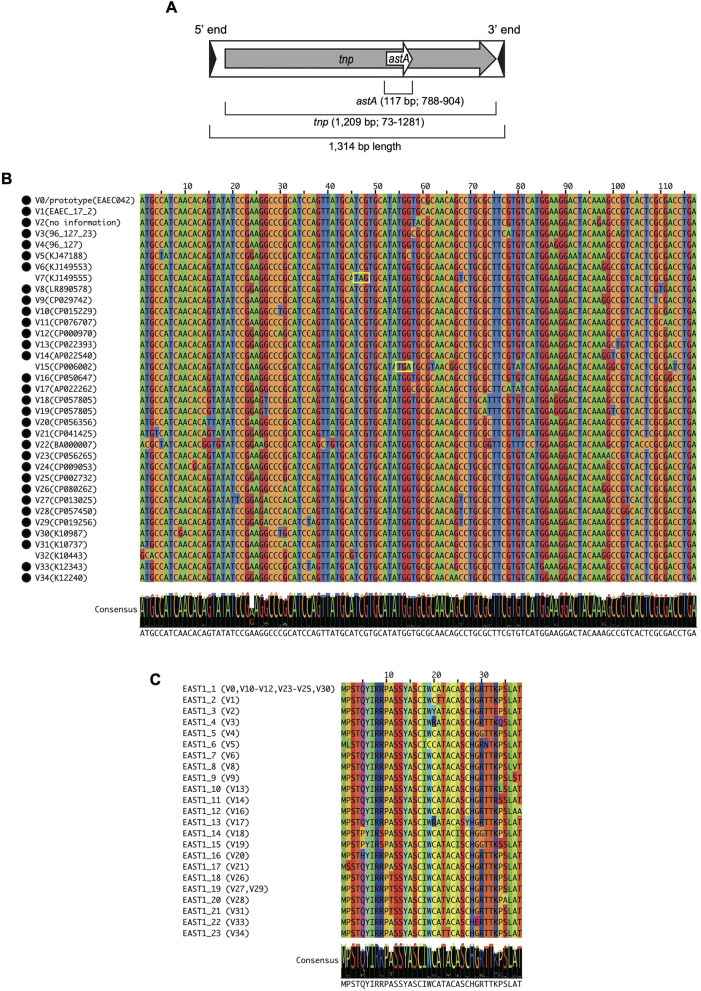
The structure of IS*1414* encoding *astA* gene **(A)**. Multiple nucleotide **(B)** and amino-acid **(C)** sequence alignments of the *astA* and EAST1 variants. Consensus sequences were shown below each sequence alignment in B and C. **(B)** The stop codon is indicated by yellow boxes and intact *astA* genes are marked with filled circles. **(C)** Variants with the same amino acid sequence are shown in parenthesis.

A notable sequence variation in the *astA* gene sequence has also been detected. Besides the prototype (referred to as V0 in this manuscript), seven variants (named V1–V7) have been identified to date (Yamamoto et al., 1997; Yamamoto and Taneike, 2000; Savarino et al., 1993; Zhou et al., 2002; Silva et al., 2014; Maluta et al., 2017). However, the biological significance of this genetic variation, the actual sequence diversity, and the prevalence of the variants have not yet been elucidated. A unique feature of the *astA* gene is that it is embedded within a transposase gene (*tnp*) of an insertion sequence (IS) IS*1414* but in its−1 reading frame (Figure 1A) (McVeigh et al., 2000). Although this was shown for the prototype *astA* gene in an enterotoxigenic *E. coli* strain 27D (McVeigh et al., 2000), it is unknown how the *astA* and its variants are associated with IS*1414* in other *E. coli* strains.

In this study, to better understand the prevalence, sequence variation, and IS association of the *astA* gene, we conducted PCR screening for the *astA* gene in 2,726 *E. coli* strains isolated from children with diarrhea in Kagoshima, Japan, and blastn search in 9,065 publicly available finished *E. coli* genomes. Using the *astA*-positive strains and *astA* genes identified through these analyses, we examined the sequence variation and IS association of *astA* and the phylogenetic distribution of the *astA* and its variants in the entire *E. coli* lineage. In addition, we report the amplification of *astA* genes associated with intact IS*1414* in multiple *E. coli* strains.

## Materials and methods

### Bacterial strains and genomic DNA preparation

For PCR screening of the *astA* gene, 2,726 *E. coli* strains isolated from stool specimens of diarrheal children who visited clinics in Kagoshima, Japan, from 2013 to 2020 (referred to as Kagoshima strains; listed in Supplementary Table 1) were used. For the blastn search of *astA*, 9,065 finished *E. coli* genomes retrieved from the NCBI database (accessed on 10 May 2022; listed in Supplementary Table 2) were used.

Template genomic DNA for PCR screening and multi-locus sequence typing (MLST) analysis was prepared by the alkaline boiling method from a 1 ml culture grown at 37°C in Lysogeny broth (nacalai tesque). Genomic DNA for whole genome sequencing was purified from a 2 ml overnight culture using the NucleoBond HMW DNA (MACHEREY-NAGEL) according to the manufacturer's instructions.

### PCR screening of the *astA* gene

Detection of the *astA* gene by PCR was performed using a primer pair (EAST1S (5′-GCCATCAACACAGTATATCC-3′) and EAST1AS (5′-GAGTGACGGCTTTGTAGTCC-3′) (Yatsuyanagi et al., 2002) and KAPATaq EXtra PCR Kit (NIPPON Genetics, Tokyo, Japan). Each reaction mixture (15 μl) contained 1 μl of template DNA, 4.5 μM of each primer and 0.3 U of polymerase. PCR was conducted with initial denaturation for 2 min at 94°C, followed by 30 cycles of 30 s at 94°C, 30 s at 55°C and 30 s at 72°C. PCR products were analyzed by agarose gel electrophoresis using 2 % Agarose S (Nippon Gene).

### Randomly amplified polymorphic DNA -PCR

RAPD-PCR was performed as described by Pacheco et al. (1997) using P1252 (5′-GCGGAAATAG-3′), P1254 (5′-CCGCAGCCAA-3′), and P1290 (5′-GTGGATGCGA-3′) primers and the KAPATaq EXtra PCR Kit (NIPPON Genetics Co., Ltd.). Each reaction mixture (25 μl) contained 5 μl of 5x KAPATaq Extra buffer, 2.5 mM MgCl_2_, 300 mM dNTPs, 1 μl of template DNA, 0.4 μM primer and 0.6 U of KAPATaq DNA polymerase. The PCR amplification steps employed were as follows: 4 cycles of 94°C for 5 min, 37°C for 5 min, and 72°C for 5 min, followed by 30 cycles of 94°C for 1 min, 37°C for 1 min and 72°C for 2 min and a final extension step at 72°C for 10 min. After PCR amplification, 10 μl of each PCR product was analyzed by agarose gel electrophoresis using 1.2% agarose S (Nippon Gene).

### Multi-locus sequence typing

MLST was performed by PCR amplification and sequencing of seven housekeeping genes (*adk, fumC, gyrB, icd, mdh, purA*, and *recA*) as previously described (Ooka et al., 2012). Alleles of each gene and the sequence types (STs) and clonal complexes (CCs) were assigned using the PubMLST (https://pubmlst.org/organisms/escherichia-spp) (Jolley et al., 2018) and the EnteroBase *E. coli/Shigella* MLST website (https://enterobase.warwick.ac.uk/) (Zhou et al., 2015), respectively.

### Whole-genome sequencing

Short-read sequencing libraries were prepared using the Nextera XT DNA Sample Prep Kit (Illumina) to obtain paired-end sequences (300 bp × 2) on the Illumina MiSeq platform. Long-read sequencing libraries were prepared using a Rapid Barcoding Kit (Oxford Nanopore Technologies) and sequenced using an R9.4.1 flow cell. After base-calling and demultiplexed using Guppy GPU v3.4.5 (Oxford Nanopore Technologies), long raw reads were filtered based on quality cut-off score of 10, and minimum length of 2,000 bp and trimmed 100 nucleotides from the start of the read using NanoFilt. A hybrid assembly was performed using microPIPE (Murigneux et al., 2021) with long and short reads with default parameters. The complete and draft genome sequences obtained in this study have been deposited in the GenBank/EMBL/DDBJ database under Bioproject no. PRJDB18009 (see Supplementary Table 2 for the list of sequenced strains and their sequencing statuses and accession numbers).

### Blastn search of the *astA* gene and assignment of new variants

The *astA* gene in the *E. coli* genomes was identified by blastn search using the known sequences of *astA* (V0–V7) (Supplementary Table 3) as queries with cutoff values of 90% nucleotide sequence identity and 60% length match. The BLAST+ source code was downloaded from the NCBI website (https://ftp.ncbi.nlm.nih.gov/blast/executables/blast+/LATEST/). New *astA* variants were defined if an identified *astA* gene showed one or more nucleotide sequence differences compared with all of the eight known sequences.

### Sequence comparison and characterization of *astA* genes, their encoding peptides, and *astA*-flanking regions

The nucleotide sequences of *astA* genes and their 1,000-bp upstream and downstream flanking regions were extracted from the genome sequences listed in Supplementary Table 2. The multiple nucleotide sequence alignments of *astA* genes and their 1,000 bp upstream and downstream regions and the amino-acid sequence alignment of *astA* gene products (EAST1 peptides) were constructed using the ClustalW function of MEGAX (Kumar et al., 2018) with default parameters. Multiple sequence alignments were visualized using JalView (V2.11.5.0) (Waterhouse et al., 2009).

### Core-gene based phylogenetic analyses, *in silico* phylo-typing, and serotyping of strains belonging to phylogroup E

The genome assemblies of 720 strains, including those of 690 strains from the NCBI database and those of 30 strains obtained in this study (18 finished and 12 draft sequences), were annotated using Prokka (Seemann, 2014), and core genes (*n* = 1,632) were identified using Roary v3.13.0 (Page et al., 2015) with 90% amino acid sequence identity cut-off. Core gene single nucleotide polymorphisms (SNPs) (*n* = 51,418) were extracted using the core gene alignment tool in Roary and used as inputs for maximum-likelihood (ML) inference with RAxML v8 (Stamatakis, 2014). The ML tree was displayed and annotated using iTOL v6 (https://itol.embl.de) (Letunic and Bork, 2016). The tree was mid-point rooted and the confidence value of each branch were estimated by bootstrap with 200 replications. Genomes showing no core gene SNPs (14 strains forming nine pairs or groups) were deduplicated (strains excluded are indicated in Supplementary Table 2). Strains used as the references for *E. coli* phylogroup assignment are also indicated in Supplementary Table 2. Serotypes of strains belonging to phylogroup E were determined by SerotypeFinder 2.0 (Joensen et al., 2015).

### Identification of other virulence-related genes in the *astA*-positive Kagoshima strains

To identify potential virulence-associated genes in each genome sequenced Kagoshima strain, we retrieved the core data set of protein sequences from the Virulence Factor Database (VFDB) website (https://www.mgc.ac.cn/VFs/). A blastx search was performed for the genome sequences using the amino acid sequences of the core dataset, with the following parameters: minimum identity of 60%, minimum coverage of 90%, and E-value cutoff of 0.01.

### Ethical approval

This study was conducted with the approval of the Ethics Committee for Epidemiological Research, Graduate School of Medical and Dental Sciences, Kagoshima University (#190105).

## Results

### Prevalence of *astA* genes in two strain sets

Of the 2,726 Kagoshima strains isolated from children with diarrhea, 185 (6.8%) were positive in the PCR screening for the *astA* gene (listed in Supplementary Table 1). In a blastn search of the *astA* gene in 9,065 finished *E. coli* genomes obtained from NCBI, 690 (7.6%) were found to possess one or more *astA* genes.

### MLST analysis of the *astA*-positive Kagoshima strains

Prior to the MLST analysis of the 185 *astA*-positive strains, we performed a RAPD analysis to identify genetically related strains among them. As 39 groups of strains with epidemiological links showed identical amplification patterns in each group (data not shown), one strain was selected from each group and used for the MLST analysis. Thus, 139 strains were subjected to the MLST analysis. This analysis revealed that they belonged to diverse STs: 63 STs were identified with ST6196 being as the largest group containing 14 strains (Supplementary Table 1). This result suggests a wide distribution of *astA*-positive Kagoshima strains in the entire *E. coli* lineage. Among the 139 *astA*-positive strains, 30 strains were selected so that they represented the phylogenetic diversity of *astA*-positive Kagoshima strains as much as possible and subjected to genome sequencing and following whole genome sequence (WGS)-based analyses.

### Sequence variation in the *astA* gene, its gene product and genomic location

By analyzing the *astA* genes in the genomes of 720 *astA*-positive *E. coli* strains (those of the 30 Kagoshima strains sequenced in this study and the 690 finished genomes obtained from the NCBI database), we identified 31 nucleotide sequence types. They included four of the eight known sequences (V1, V2, V3, and V5 were not detected in this strain set) and 27 newly identified types which were named V8–V34. In addition, various lengths of *astA* fragments that encode only C-terminal parts of EAST1 were detected in 350 genomes.

As shown in Figure 1B, while many of the variants (23/34 variants) showed 1- or 2-bp difference compared with the V0/prototype, the remaining 11 variants showed 3–12 bp difference. Of these variants, four variants were inactivated by premature stop codons (V7 and V15) or base-changes in the start codon (V22 and V32). Amino-acid sequence alignment of the 31 *astA* genes that encode full-length EAST1 (Figure 1C) revealed that seven variants encoded EAST1 identical to that encoded by the V0/prototype. In addition, two variant *astA* genes encoded an identical EAST1 peptide. Thus, 23 variants of EAST1 were identified. We named them EAST1_1 - EAST1_23, of which EAST1_1 corresponds to the EAST1 encoded by the V0/prototype *astA* gene. Although three EAST1 types contained three, four or five amino-acid substitution compared to the EAST1_1, the remaining types showed one or two amino-acid differences. Of the 38 amino acid residues, 22 were fully conserved, including two of the four cysteine residues present in EAST1_1. These four cysteine residues (Cys-17, 20, 24, and 27) are involved in the formation of two disulfide bridges responsible for the heat stability and, especially, the Cys-17 residue is important for a disulfide bridge integrity for toxicity expression (Uzzau and Fasano, 2000). Of these four cysteine residues, Cys-17 is conserved in all EAST1 types, but in EAST1_3, 4, and 13, one or two amino-acid substitutions occurred at Cys-20 and Cys-27.

Among the 720 strains analyzed, the most dominant nucleotide sequence type was V22 (217 strains; 30.1%), followed by V0/prototype (146 strains; 20.3%), V6 (68 strains; 9.4%), V27 (46 strains; 6.4%), and V12 (36 strains; 5.0%). The other types were detected in less than 2% of the 720 strains (Table 1). Of the top five types, V22 has been inactivated as mentioned above. V12 encoded the EAST1 identical to that encoded by V0/prototype (EAST1_1). As additional seven variants encoded EAST1_1, the most predominant EAST1 type was EAST1_1, which can be potentially produced by 199 strains.

**Table 1:** Prevalence and localization of *astA* variants in 720 *E. coli* strains analyzed.

***astA* variant type**	**Number of strains**	**Localization**			**Number of strains with multicopies**	**Maximum copies**
		**Chromosome**	**Plasmid**	**Both**		
V0/prototype	146	60	73	13	43	11
V1	0	0	0	0	0	0
V2	0	0	0	0	0	0
V3	0	0	0	0	0	0
V4	1	0	1	0	0	1
V5	0	0	0	0	0	0
V6	68	64	4	1	1	3
V7	12	12	0	0	0	1
V8	1	0	1	0	0	1
V9	5	0	5	0	0	1
V10	1	1	0	0	0	1
V11	2	0	2	0	0	1
V12	36	36	0	0	0	1
V13	2	2	0	0	0	1
V14	9	9	0	0	0	1
V15	9	0	9	0	1	2
V16	2	0	2	0	0	1
V17	2	0	2	0	0	1
V18	3	0	3	0	1	2
V19	2	0	2	0	0	1
V20	1	0	1	0	0	1
V21	4	1	3	0	1	2
V22	217	214	3	0	1	2
V23	2	0	2	0	2	2
V24	7	0	7	0	0	1
V25	4	0	4	0	0	1
V26	1	0	1	0	0	1
V27	46	45	1	0	0	1
V28	2	2	0	0	0	1
V29	9	9	0	0	0	1
V30	1	1	0	0	0	1
V31	1	1	0	0	0	1
V32	5	4	1	0	0	1
V33	1	1	0	0	0	1
V34	1	0	1	0	0	1
fragment	350	-	-	-	-	-

As for the genomic locations of the *astA* genes identified in the 720 strains, they were located on chromosome or plasmid. While the locations of the *astA* genes other than V0/prototype showed some bias toward either chromosome or plasmid, V0/prototype was located almost evenly on chromosome and plasmids and 12 strains carried it on both chromosome and plasmid (Table 1). As *astA*-bearing plasmids were 28-380 kb in size (data not shown), they are likely single- or low-copy plasmids and many of them are probably transmissible or were previously transmissible.

### Strains possessing multiple types of *astA* genes and multiple copies of the V0/prototype gene

Interestingly, 28 strains harbored two or three types of potentially active *astA* genes (encoding a full length EAST1) with various combinations: two types in 27 strains and three types in one strain (Table 2). Of the 15 combinations detected, V0/prototype involved in eight combinations, which was an expected finding from its wide distribution. A more interesting and important finding was that a notable number of V0/prototype-containing strains (43/146) possessed multiple copies of this type of *astA* gene (Table 1). Of these strains, while 22 strains possessed two copies and seven strains possessed three copies, 14 strains contained five or more copies (up to 11 copies). This was in sharp contrast to other types of *astA* genes: only six variants, of which two were inactivated ones (V15 and V22), appeared twice or three times in the genomes of only eight strains. These findings indicate that the amplification of *astA* occurred almost specifically for the V0/prototype.

**Table 2:** Strains harboring multiple active *astA* genes.

**Multiple variants**	**Number of strains**
V0/prototype, V27	9
V6, V27	2
V6, V12	2
V18, V19	2
V6, V9	2
V0/prototype, V6	2
V0/prototype, V9	1
V0/prototype, V14	1
V0/prototype, V18	1
V0/prototype, V25	1
V0/prototype, V26	1
V6, V29	1
V12, V14	1
V17, V30	1
V0/prototype, V6, V9	1
Total	28

### IS*1414*-association of *astA* genes

To investigate how various types of *astA* identified in the 720 strains are associated with IS*1414*, we analyzed the sequences flanking each *astA* gene (1,000-bp sequences upstream and downstream of *astA*). This analysis revealed that while all were associated with IS*1414*, many of the associated IS*1414* have been decayed by deletion or inactivated by mutations in the transposase gene (Supplementary Figures 1, 2). However, most V0/prototype genes (203/270) were associated with intact IS*1414*, thus they are transposable as a part of IS element (Supplementary Table 2). The V30 and V31 genes were also associated with intact IS*1414*, but they were harbored by only one strain, respectively.

### Phylogenetic view of the 720 a*stA*-positive *E. coli* strains and the distribution of V0/prototype and variant *astA* genes

**Figure 2:**
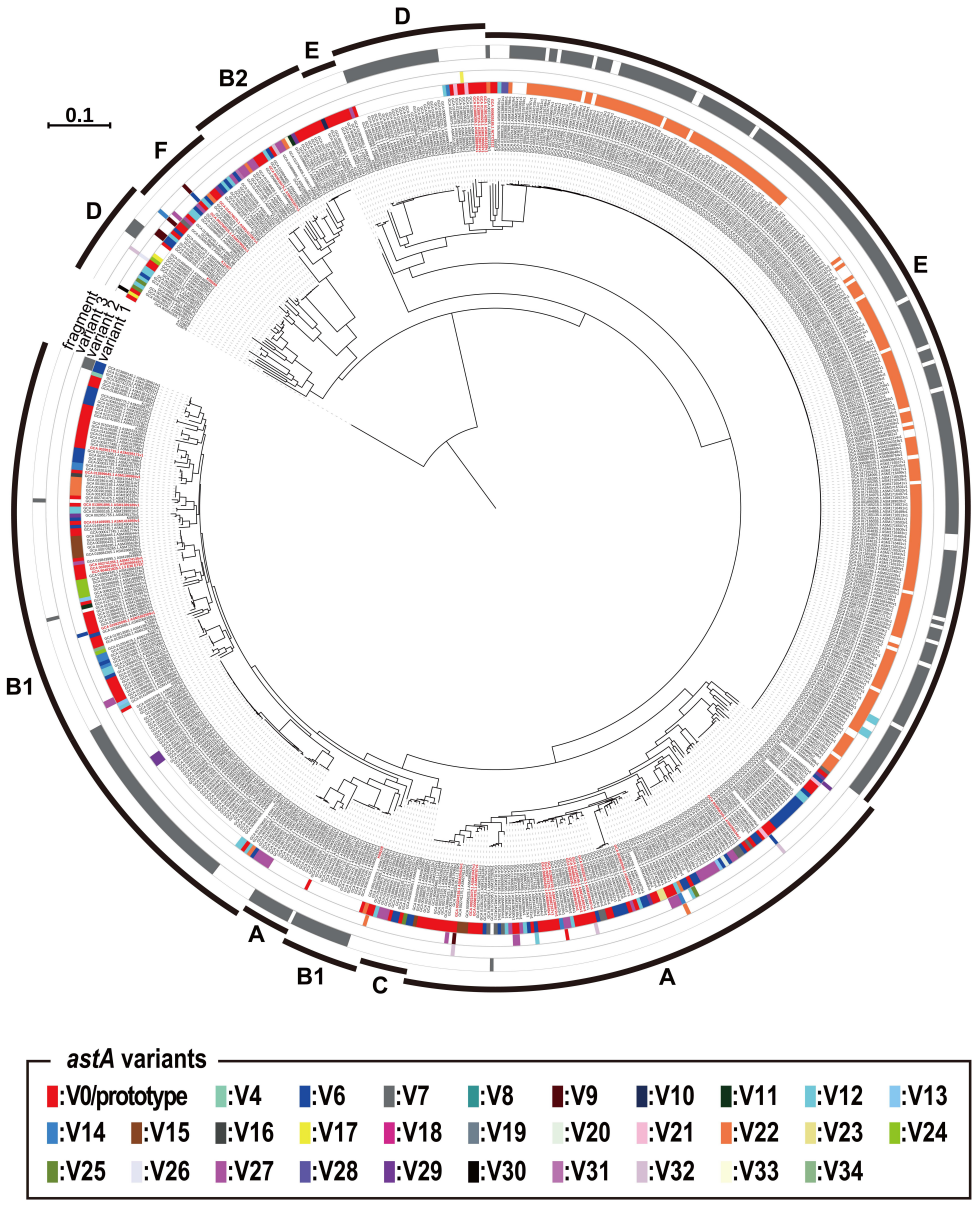
Phylogenetic view of the 720 *astA*-positive *E. coli* strains that have been genome sequenced and the distribution of the 35 *astA* variants and the *astA* fragment of in these strains. The types of variants carried by each strain are indicated as variant 1, variant 2, and variant 3. For example, strains carrying three types such as V0, V3, and V5 have each type shown under variant 1, 2, and 3, respectively. Strains that possess short *astA* gene fragments, which are insufficient to determine the variant type, are indicated in gray as “fragment”. The strains possessing multiple copies of the V0/prototype *astA* gene are indicated in red.

We constructed an ML phylogenetic tree of the 720 a*stA*-positive strains based on their core gene sequences to investigate the phylogenetic relationship of the 720 strains and the distribution of the V0/prototype and variant *astA* genes and *astA* fragments in this strain set. As shown in Figure 2, the *astA* genes were distributed in all *E. coli* phylogroups. Notably, most of V22 (the most prevalent but inactivated variant) were distributed in phylogroup E: 94.5% of V22-positive strains belonged to this phylogroup (Supplementary Table 4). However, this bias was apparently introduced by the presence of a large number of O157:H7 strains in the strain set analyzed: it included 258 O157:H7 strains, of which 202 contained the V22 variant (Supplementary Table 5). Except for this bias of V22, there was no clear association between the *astA* type and the lineage (phylogroup) of strains. For example, the V0/prototype *astA* gene was distributed in all phylogroups and the strains harboring multiple copies of V0/prototype were also found in all phylogroups.

### Correlation between the type and copy number of the *astA* gene and the severity of diarrhea

We investigated the relationship between clinical symptoms and *astA* gene variants in 30 *Escherichia coli* isolates collected in Kagoshima Prefecture that were subjected to whole-genome sequencing in this study. Detailed epidemiological data were available for 14 of the 30 patients. However, isolates of *Campylobacter jejuni* or norovirus were also detected in 8 of these cases, suggesting that *E. coli* was not the primary causative agent; thus, these cases were excluded from further analysis. In the remaining 6 cases, only *astA*-positive *E. coli* strains were isolated. Among these, five isolates—excluding strain K9291—harbored intact *astA* variants (V0/prototype or V33), while K9291 carried the inactive V15 variant. Furthermore, a virulence factor search against the VFDB database revealed that only strain K12343 did not possess any potential virulence factors other than the *astA* gene. The remaining strains carried additional virulence factors, including *senB* (encoding enterotoxin), the *cfa* operon (encoding CFA/I fimbriae), or genes encoding effector proteins (EspL, EspR, EspX, EspY) secreted via the locus of enterocyte effacement-encoded type III secretion system (LEE-T3SS). However, none of the five strains possessed the LEE region itself in their genomes, suggesting that the LEE-T3SS is absent and therefore the effector proteins are unlikely to be secreted (Supplementary Table 6).

## Discussion

The results of our analyses of two sets of *E. coli* genomes indicate that the frequency of *astA*-positive *E. coli* strains is about 7% both in the 2,726 strains isolated from children with diarrhea in Kagoshima, Japan, and the 9,065 finished *E. coli* genomes obtained from NCBI, most of which were the genomes of non-Japanese strains. In previous studies, the detection rate of the *astA* gene in animal-, healthy human-, and diarrheal patient-derived *E. coli* strains was 20.7–86.8%, 2.4–20.5%, and 4.8–11.2%, respectively, although the number of samples and detection methods were different between the studies (Fujihara et al., 2009; Wang et al., 2017; Sukkua et al., 2017; Awad et al., 2020). Thus, the detection rate in the Kagoshima strains (diarrheal patient-derived *E. coli* strains) was in a range similar to those of the previous studies.

Our analysis of the *astA* genes identified in this study added 27 novel sequence variants of *astA* (V8–V34) to the previous list of *astA* sequences (V0/prototype and variants V1–V7), revealing the notable sequence diversity of *astA* (Figure 1B). Importantly, four of the 35 types of *astA* have been inactivated by premature stop codons or mutations in start codon (Figure 1B). In particular, one of the four variants was the most prevalent *astA* type, V22, which was found in 217 strains out of the 720 strains analyzed (Table 1). Moreover, *astA* fragments of various lengths were found in as many as 350 strains. These findings indicate the need of distinguishing strains harboring functional and non-functional *astA* genes when considering the contribution of EAST1 as a virulence factor. As PCR protocols currently used cannot distinguish them (Yamamoto and Echeverria, 1996; Yatsuyanagi et al., 2002), novel methods that specifically detect functional *astA* genes detect need to be developed.

At the amino-acid sequence level, 23 types of EAST1 (EAST1_1 to EAST1_23) were identified. EAST1_1 encoded by V0/prototype (second most prevalent) and seven variant *astA* genes were most predominant. Most of other EAST1 types contained one or two amino-acid substitutions. However, in four EAST1 types, substitutions occurred at one or two cysteine residues which may be important for the function as a heat-stable enterotoxin. The functions of these EAST1 types as well as other EAST1 types containing any amino-acid substitution(s) also need to be examined to understand the contribution of EAST1 as a virulence factor.

There are several studies showing conflicting results regarding the correlation between the copy number of the *astA* gene and toxicity (McVeigh et al., 2000; Ruan et al., 2012), but a clear conclusion has yet to be established. Although the correlation between the copy number of the *astA* gene and toxicity remains unclear, it is well established that increases in the copy number of virulence-related genes can enhance pathogenicity in other bacterial species. In *Vibrio cholerae*, the *ctx* operon encoding cholera toxin forms tandem repeats through gene duplication, and strains with higher numbers of repeats exhibit increased virulence (Mekalanos, 1983). Additionally, in *Yersinia enterocolitica*, it is known that pathogenicity is enhanced during infection by increasing the copy number of a plasmid encoding a type III secretion system (Wang et al., 2016). Although the further functional analyses are required to understand a potential of virulence of the *astA* gene, if the copy number of *astA* gene correlates positively with virulence, then isolates carrying multiple copies of V0/prototype are of particular importance because they included strains carrying more than five copies (up to 11 copies) (Table 2).

The emergence of these strains carrying multiple copies of V0/prototype *astA* gene is apparently linked to the fact that most of the V0/prototype are associated with intact IS*1414* (Supplementary Table 2), and thus, they can be amplified on the genome upon the transposition of IS*1414*. It should also be emphasized that all types of *astA* genes are associated with IS*1414*, but the IS*1414* elements associated with the *astA* genes other than V0/prototype and two minor variants (V30 and V31) are currently inactive due to various deletions or the mutations in their transpose genes. A notable number of prototype-bearing strains (43/146 strains) possessed multiple copies (two to 11 copies) of this type of *astA* gene, indicating that the amplification has predominantly occurred in the prototype, which was driven by IS*1414* amplification. However, given that the IS*1414* associated with V30 and V31 also remain structurally intact, it is plausible that similar amplification events may occur in these variants in the future. Frequent structural alterations of IS*1414*, such as deletion and point mutations, are likely responsible for the generation of *astA* gene fragments of different lengths, found in as many as 350 strains.

Since all *astA* variants are encoded within IS*1414*, it is suggested that IS*1414* may be involved in the wide distribution of *astA* genes in almost all the *E. coli* lineages (Figure 2). It also resulted in the variable genomic locations of *astA*, either or both of chromosome and plasmids. As the IS*1414*-bearing plasmids are large plasmids, many of them are probably transmissible (or were transmissible before). Thus, these plasmids also likely contributed to the spread of *astA*.

Based on the analysis of six cases in which only *E. coli* strains harboring the *astA* gene were isolated, we found that strain K12343, which carries an intact V33 variant, did not possess any additional virulence-associated genes. This suggests the possibility that EAST1 may be directly involved in the diarrheal symptom in this case. In contrast, the four strains (K9228, K9910, K11627, and K12196) carrying the V0/prototype type also harbored other virulence-associated factors, making it difficult to conclude that the V0/prototype type alone was directly responsible for the diarrheal symptoms.

In conclusion, our screening of two large *E. coli* strain sets, followed by detailed analyses of the *astA* gene and *astA*-positive strains, revealed several important findings: (i) notable sequence diversity in *astA* gene and its gene product, EAST1; (ii) the presence of several non-functional *astA* variants; (iii) widespread distribution of *astA* gene fragments; (iv) a strong association between the V0/prototype *astA* genes and intact IS*1414*, which have led to amplification of this type of *astA* gene in some strains; and (v) broad dissemination of the *astA* gene in almost all the *E. coli* lineages, likely driven by IS*1414*-mediated transposition. Furthermore, since the IS*1414* associated with variants V30 and V31 also remain structurally intact, it is plausible that similar amplification events could occur in these variants in the future. These findings will be an important basis to investigate the virulence of *astA*-positive strains and the role of EAST1 as a virulence factor.

## Data Availability

The datasets presented in this study can be found in online repositories. The names of the repository/repositories and accession number(s) can be found in the article/Supplementary material.
