# Supplementary material for: Prevalence, sequence diversity, and amplification of an IS-associated enterotoxin gene, astA, in Escherichia coli
Source: Front Microbiol. 2025 Oct 22;16:1635769. doi: 10.3389/fmicb.2025.1635769 (PMC12585946; doi:10.3389/fmicb.2025.1635769)
Supplement: Supplementary file 2 [file Data_Sheet_1.pdf]

Figure legends for Supplemental Figures

Supplemental Figure 1. Structural differences of the *astA* variant-encoded IS*1414*. Defective genes are indicated by dotted line.

Supplemental Figure 2. Multiple sequence alignments of the 1,000 bp upstream and downstream regions of the 35 *astA* variants. More than 50% conserved sequences are shaded black. The IS*1414* regions are framed in red. The coding regions for transposase and EAST1 are indicated by blue and purple, respectively. Insertion or deletion and the premature stop codon in IS*1414* regions are indicated by blue boxes.
