# Supplementary material for: Prevalence, sequence diversity, and amplification of an IS-associated enterotoxin gene, astA, in Escherichia coli
Source: Front Microbiol. 2025 Oct 22;16:1635769. doi: 10.3389/fmicb.2025.1635769 (PMC12585946; doi:10.3389/fmicb.2025.1635769)
Supplement: Supplementary file 3 [file Data_Sheet_2.pdf]

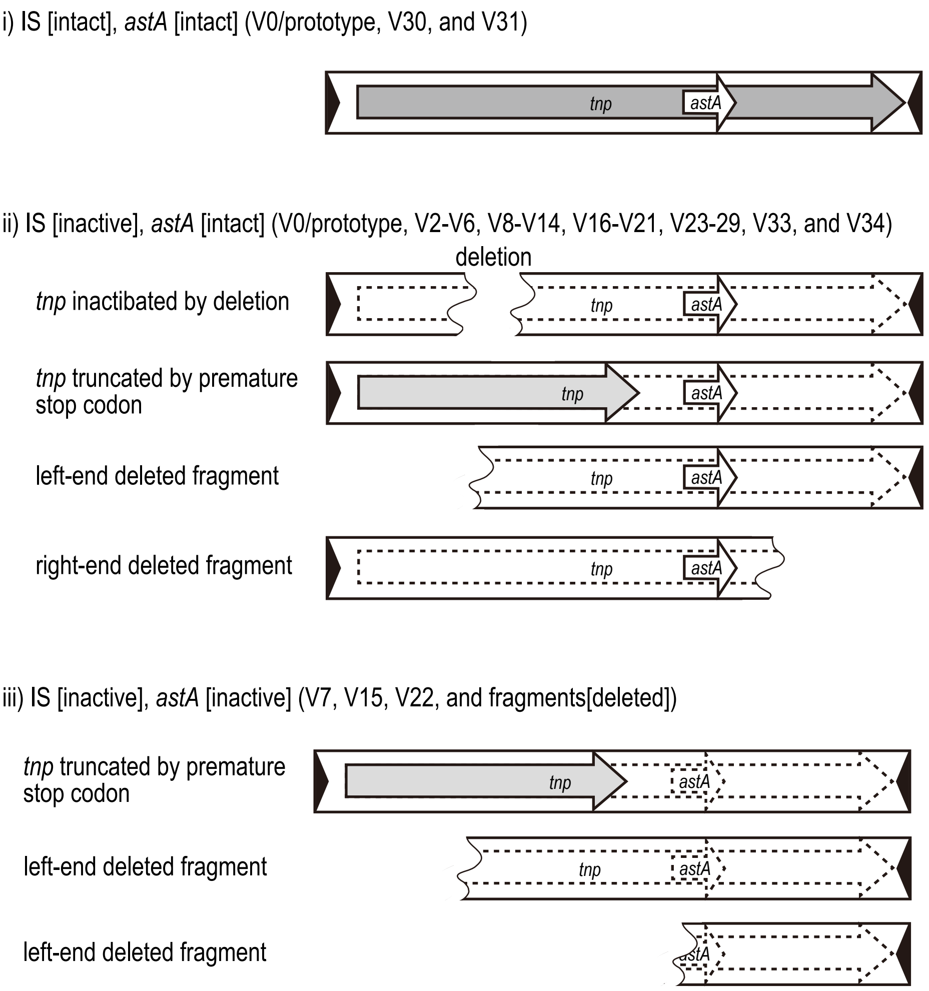
i) IS [intact], astA [intact] (V0/prototype, V30, and V31)
tnp
astA
ii) IS [inactive], astA [intact] (V0/prototype, V2-V6, V8-V14, V16-V21, V23-29, V33, and V34)
deletion
tnp inactibated by deletion
tnp truncated by premature stop codon
left-end deleted fragment
right-end deleted fragment
iii) IS [inactive], astA [inactive] (V7, V15, V22, and fragments[deleted])
tnp truncated by premature stop codon
left-end deleted fragment
left-end deleted fragment
